# Pancreatic stellate cells support human pancreatic β-cell viability *in vitro* and enhance survival of immunoisolated human islets exposed to cytokines

**DOI:** 10.1016/j.mtbio.2024.101129

**Published:** 2024-06-18

**Authors:** Tian Qin, Shuxian Hu, Defu Kong, Jonathan R.T. Lakey, Paul de Vos

**Affiliations:** a Immunoendocrinology, Division of Medical Biology, Department of Pathology and Medical Biology, University of Groningen and University Medical Center Groningen, Hanzeplein 1, EA 11, 9713 GZ, Groningen, the Netherlands; b Biological and Environmental Engineering, Cornell University, Ithaca, NY, 14853, USA; c Department of Liver Surgery, Renji Hospital, School of Medicine, Shanghai Jiao Tong University, Shanghai, 200127, China; d Department of Surgery, University of California Irvine, Irvine, CA, 92868, USA; e Department of Biomedical Engineering, University of California Irvine, Irvine, CA, 92697, USA

**Keywords:** Islet transplantation, Pancreatic beta cell, Pancreatic stellate cell, Type 1 diabetes

## Abstract

Pancreatic islet transplantation is proposed as a cure for type 1 diabetes mellitus (T1D). Despite its success in optimal regulation of glucose levels, limitations in longevity of islet grafts still require innovative solutions. Inflammatory stress post-transplantation and loss of extracellular matrix attribute to the limited β-cell survival. Pancreatic stellate cells (PSCs), identified as pancreatic-specific stromal cells, have the potential to play a crucial role in preserving islet survival. Our study aimed to determine the effects of PSCs co-cultured with human CM β-cells and human islets under inflammatory stress induced by a cytokine cocktail of IFN-γ, TNF-α and IL-1β. Transwell culture inserts were utilized to assess the paracrine impact of PSCs on β-cells, alongside co-cultures enabling direct interaction between PSCs and human islets. We found that co-culturing PSCs with human CM β-cells and human cadaveric islets had rescuing effects on cytokine-induced stress. Effects were different under normoglycemic and hyperglycemic conditions. PSCs were associated with upregulation of β-cell mitochondrial activity and suppression of inflammatory gene expression. The rescuing effects exist both in indirect and direct co-culture methods. Furthermore, we tested whether PSCs have rescuing effects on human islets in conventional alginate-based microcapsules and in composite microcapsules composed of alginate-pectin collagen type IV, laminin sequence RGD, Nec-1, and amino acid. PSCs partially prevented cytokine-induced stress in both systems, but beneficial effects were stronger in composite capsules. Our findings show novel effects of PSCs on islet health. Islets and PSCs coculturing or co-transplantation might mitigate the inflammation stress and improve islet transplantation outcomes.

## Graphical abstract

**Figure:**
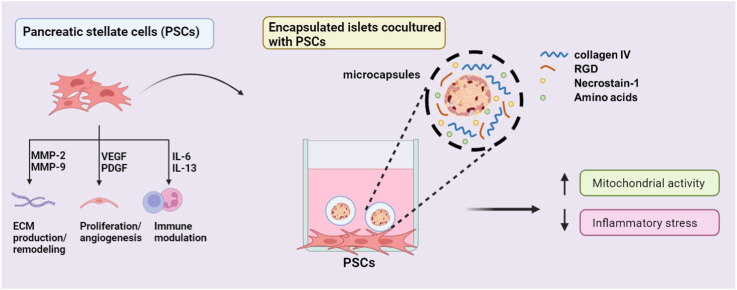

## 1 Introduction

Pancreatic β-cells are destroyed as a consequence of an autoimmune reaction in patients with Type 1 diabetes (T1D). Consequently, T1D individuals are unable to generate endogenous insulin in response to fluctuating blood glucose levels [1]. T1D patients require daily insulin injections to regulate glucose levels but this therapy cannot prevent regular hyper- and hypoglycemic events, which impact the quality of life and can also lead to secondary and progressive diabetic complications.

Individuals with T1D would benefit from a therapy that controls blood glucose levels minute by minute within normal ranges [2]. This can be achieved by introducing an endogenous insulin source such as an islet transplant. Islet transplantation is a procedure that requires minor surgery and has been shown to enable real-time glucose control. By transplanting islets, a cluster of several cell types including insulin- and glucagon-secreting cells, precise blood glucose control can be achieved [3]. However, the need for systemic chronic immunosuppression as well as donor shortages restricts the availability of the procedure to only a small group of individuals with T1D, which are usually patients with end-stage renal failure or with brittle diabetes [4].

During the past decade, technologies have been developed that might lead to more widespread application of islet transplantation. The introduction of new stem cell differentiation protocols and gene editing technologies such as CRISPR-Cas9 in pigs [5] has brought the application of both transgenic xenotransplantation and stem-cell derived islets close to clinical application [6]. This could significantly address the issue of donor shortage for transplantation. Additionally, new approaches for immunoisolation have demonstrated successes of up to a year of insulin independence in experimental animals [7], providing hope for immunosuppressive-free transplantation of islets.

To obtain long-term functioning immunoisolated islet grafts, it is essential to make modifications both on the outside and inside of the capsules [7]. Examples of modifications on the outside of capsules include the adherence of antifibrotic factors or immunomodulating molecules such as pectins [8,9]. Modifications inside the capsules are especially needed to prevent the loss of islet-cells under cytokine stress. Many islets suffer damaging effects and may die in immunoisolating devices such as alginate-based microcapsules directly after implantation due to the release of various cytokines by the host [10]. This may lead to the loss of up to 60 % of the transplanted islet graft in the immediate period after transplantation [7,11]. We have previously shown that incorporating specific extracellular matrix (ECM) components, such as collagen type IV and RGD (arginine-glycine-aspartic acid), or other survival factors into the intracapsular environment can extend the functional survival of islets [9,11,12]. However, more might be needed for long-term survival.

The strategy for modifying the intracapsular environment is to enhance the longevity of encapsulated islet grafts by mimicking the pancreatic microenvironment. The pancreatic microenvironment orchestrates a complex interplay of various cell types and signals [13], each contributing uniquely to the delicate balance necessary for the maintenance of pancreatic function and support of islet survival. Among these, pancreatic stellate cells (PSCs) are emerging as central players in the regulation of cellular dynamics within the pancreas. PSCs transform from quiescent cells to activated myofibroblast-like cells through various stimuli [14], where activated PSCs secrete cytokines, ECM molecules and growth factors that promote tumor cell proliferation and invasion and alleviate immune response. Remarkably, there has been a notable lack of focus on PSCs in healthy non-cancerous situations where PSCs also produce ECM and create a favorable microenvironment for islet cells.

The use of pancreatic stellate cells (PSCs) to support human pancreatic β-cell viability represents a new approach for support of pancreatic β-cell-transplants. PSCs are intrinsic stromal cells within the pancreas and support islet health, which suggests that PSCs may have similar or even more specific effects as for example the commonly applied approach of including mesenchymal stromal cells (MSCs) in islet grafts. PSCs, when activated, secrete a variety of growth factors, cytokines, and ECM components that can create a supportive environment for β-cells [15]. This supportive role of PSCs is crucial, especially in the context of inflammatory cytokine exposure that typically follows after islet transplantation. The effects of PSCs on pancreatic β-cells either under normal conditions or inflammatory stress remain to be elucidated. By leveraging the natural supportive properties of PSCs, we can enhance the survival and function of transplanted β-cells, potentially reducing the need for immunosuppressive therapy and improving transplant outcomes.

The current study was undertaken to determine whether activated PSCs have a beneficial impact on the viability of isolated human pancreatic β-cells. This was done both in an *in vitro* setting by using *in vitro* co-culture systems (Transwell) in which PSCs were in direct or indirect contact with isolated human β-cells. Furthermore, we tested whether PSCs have rescuing effects on human islets in conventional alginate-based microcapsules and in composite microcapsules composed of alginate-pectin collagen type IV, laminin sequence RGD, Nec-1, and amino acid. Possible rescuing effects were tested in the absence and presence of the inflammatory cytokines: interferon-γ (IFN-γ), tumor necrosis factor-α (TNF-α) and interleukin 1-β (IL-1β). The intrinsic expression of immune and angiogenesis related genes were studied under the mentioned circumstances. Our data demonstrates a unique role of activated PSCs on human β-cells under normal culture conditions and in an inflammatory environment.

## 2 Materials and methods

### 2.1 Chemicals

Chemicals were obtained from Sigma Aldrich (St. Louis, MO, USA). Cell culture materials were obtained from Lonza (Basal, Switzerland) unless otherwise stated. The cytokines IFN-γ, TNF-α and IL-1β were obtained from ImmunoTools, Friesoythe, Germany.

### 2.2 CM human β-cells

The CM human β**-**cell line [16] grows routinely as an adherent semiconfluent monolayer. Cells were cultivated in 5 % CO_2_/95 % humidified air at 37 °C in standard RPMI1640 medium supplemented with 5 % fetal calf serum (FCS), 2 mM glutamine, 10 000 units penicillin+10 mg/mL streptomycin and 11 mM glucose [16].

β-cells were routinely grown at 11 mM glucose. To make the CM cell line more sensitive to glucose stimulation, cells were prepared at either conventional glucose levels (11 mM) or at low glucose level (0.8–1.0 mM) [17]. Cells for low glucose levels were split and gradually transferred in medium containing lower concentrations of glucose until the culture was settled at 0.8–1 mM glucose. CM cells were then cultured for another 8 weeks before being used in the experimental studies.

### 2.3 Primary human pancreatic stellate cells (PSCs)

Primary human PSCs (HPSCs; ScienCell, Carlsbad, CA, USA; Cat #3830, Lot #27648) were isolated from human pancreata and were maintained in Stellate cell medium (SteCM, Cat #5301, ScienCell, Carlsbad, CA, USA) supplemented with stellate cell growth supplement (ScienCell, Carlsbad, CA, USA Cat #5352), 2 % FCS (ScienCell, Carlsbad, CA, USA; Cat #0010) and 1 % penicillin-streptomycin (ScienCell, Carlsbad, CA, USA Cat #0503), in an incubator at 37 °C at an atmosphere of 5 % CO_2_. All experiments were carried out at low cell passage (<P3).

### 2.4 Human islet isolation and culture

Human pancreatic islets were isolated from cadaveric pancreata at the 10.13039/501100005039Leiden University Medical Center and the European Consortium for Islet Transplantation (provided through the 10.13039/100022690JDRF award 31-2008-416, Islet for Basic Research program, Milan, Italy), as previously described [18]. The detailed information of islet and donor data are mentioned in Table 1. All the procedures were approved and carried out in accordance with the code of proper secondary use of human tissue for research in The Netherlands as formulated by the Dutch Federation of Medical Scientific Societies.

**Table 1:** Islet and donor information.

Age	Gender	BMI	Blood type	Islet isolation center	Cause of death	Estimated purity (%)	Estimated viability (%)
60	M	25	O	LUMC a	non cardiac	30	>80
52	M	25	O	LUMC	non cardiac	60	>80
57	F	14	A	LUMC	non cardiac	60	>80
46	M	25	A	LUMC	Euthanasia	90	>80
66	F	31	O	LUMC	subarachnoid hemorrhage	80	>80
79	M	26	O	LUMC	non cardiac	75	>80

a LUMC: Leiden University Medical Center. BMI: body mass index; Estimated purity: (% of islet tissue versus exocrine tissue); Estimated viability: % of viable islets by fluorescein diacetate–propidium iodide (FDA-PI) staining.

After shipment to the University Medical Center Groningen, islets were cultured in CMRL-1066 (Gibco, Bleiswijk, the Netherlands), supplemented with 10 % fetal bovine serum, 50 U/mL penicillin, and 50 μg/mL streptomycin, as previously described [18].

### 2.5 Microencapsulation and co-culture

Alginate and DM18 pectin powder were dissolved in Ca^2+^ Krebs-Ringers-Hepes (KRH) with an osmolarity of 220 mOsm [19]. Human islets were encapsulated in 1 % w/v DM18 pectin with 3 % w/v alginate solution or in 3.4 % w/v alginate solution which served as control group. In the composite group, an alginate/pectin mix solution was supplemented with 50 μg/mL collagen type IV, 10 μM RGD, 100 μM Nec-1, amino acids (10 mM alanine and glutamine, 20 mM glycine).

The solutions mentioned above were then mixed with human islets at a concentration of 1000 islets/mL. Droplets were produced with an air-driven droplet generator as previously described [12]. The droplets were transformed into microcapsules by collecting droplets in a 100 mM CaCl_2_ (10 mM HEPES, 2 mM KCl) solution and allowing 10 min of gelification. All the solutions were sterilized by 0.2 μm filtration. All droplets were washed with KRH buffer containing 25 mM CaCl_2_ for at least 3 times. Islet containing alginate capsules were handpicked and used in further experiments.

PSCs (passage 2/3) were plated at a density of 5 × 10^4^/mL in 12 or 24-well culture plates and cultured for 24 h prior to coculturing with CM β-cells or islets. (a) CM β-cells were co-cultured with PSCs, CM β-cells were seeded at a density of 1 × 10^5^/ml in the Transwell inserts (Corning Inc., Corning, NY, USA) with 0.4 μm pore size, which were put on top of the PSCs, with or without cytokines in culture medium. (b) Pancreatic islets (50 islets for 12-well plate or 25 islets for 24-well plate) were cultured and then placed on top of the PSCs with Transwell inserts or direct contact, with or without cytokines in the culture medium. (c) Microencapsulated islets (50 capsules for 12-well plate or 25 capsules for 24-well plate) were put on top of PSCs with or without cytokines in culture medium. This was done without the Transwell inserts to allow PSCs and islets to interact.

### 2.6 Viability

A WST-1 (Roche, Indianapolis, IN, USA) assay was applied to investigate the effect of PSC co-culture on β-cell mitochondrial activity. The CM β-cells or human islets, alone or co-cultured with PSCs, were seeded in 24-well plates with or without cytokine exposure. After 24h incubation, WST-1 reagent was added at 1:10 of culture medium to each well. Wells without cells were used as blank control. After 60 min incubation at 37 °C, the absorbance was measured at 450 nm (reference at 650 nm) using a BioTek Epoch 2 microplate reader. Results were normalized to total DNA content as was determined using the Quanti-iT PicoGreen dsDNA kit (Invitrogen, USA). Results were expressed as percentage of the control.

### 2.7 In vitro glucose-stimulated insulin secretion (GSIS) test

Human pancreatic islets were tested for GSIS after co-culture with PSCs for 24h. Briefly, 25 human islets were handpicked and transferred to glass incubation tubes. The first incubation consisted of a low glucose concentration (2.75 mM) solution in KRH buffer containing 25 mM CaCl_2_ for 1 h, followed by a high glucose concentration (16.7 mM) buffer in KRH for 1 h, and another 1-h incubation in 2.75 mM glucose in KRH. At the end of each incubation, the supernatants were removed for further insulin measurements via Enzyme-Linked Immunosorbent Assay (ELISA) (Mercodia AB, Sweden) using a spectrophotometric plate reader according to the manufacturer's protocol. DNA content of islets was quantified with a fluorescent Quant-iT PicoGreen double-strand DNA assay kit (Invitrogen, USA). The insulin secretory responses were normalized to total DNA content and expressed as mU L^−1^ μg^−1^ DNA hour^−1^.

### 2.8 Immunofluorescence staining

For immunofluorescence, islets and PSCs were cultured on coverslips and fixed for 15 min with 4 % paraformaldehyde (Merck, Darmstadt, Germany) and permeabilized with 0.3 % Triton X100 (Merck) for 30 min. Non-specific binding was blocked by incubating the cells with 2 % BSA (Sigma-Aldrich) for 30 min. Then cells were incubated overnight at 4 °C with the primary antibodies of insulin, α-SMA, vimentin, desmin, GFAP, CK19, and CD68. Subsequently, the sections were incubated with appropriate secondary antibodies for 30 min (Supplementary Table 1). Afterwards, the islets and PSCs were washed with PBS for 10 min and sealed with Antifade Mounting Medium with DAPI (Vector laboratories, CA, USA). The staining was analyzed using a Zeiss 410 inverted laser scan microscope (Leica Microsystems, Wild Heerbrugg, Germany). Data were processed using ImageJ software (Version 1.52; National Institutes of Health, Bethesda, MD, USA).

### 2.9 Quantitative reverse-transcription polymerase chain reaction (qRT-PCR)

A qRT-PCR was performed to determine the gene expression in pancreatic islets or PSCs. Basically, cells were homogenized with TRIzol reagent (Life Technologies, Carlsbad, CA, USA). Total RNA was isolated following the manufacturer's instructions. Reverse-transcribed cDNA was subsequently obtained using SuperScript II Reverse Transcriptase (Invitrogen, USA). qPCR was performed with a FastStart Universal SYBR-Green Master kit (Roche, Indianapolis, IN, USA) for the genes listed below (primer sequences are listed in Table S2). Reactions were carried out in 384-well PCR plates (Thermo Scientific) with a ViiA7 Real-Time PCR System (Applied Biosystems, Carlsbad, CA, USA). Delta Ct values were calculated and normalized to the housekeeping gene β-actin. The 2^-△△Ct^ method was used for the comparative quantification of gene expression of the genes.

### 2.10 ELISA

Quantikine ELISA Kits (R&D systems, Minneapolis, USA) for human and rat IL-6 and VEGF was performed according to the manufacturer's instructions using a microplate spectrophotometer BioTek Epoch 2 at 450 nm with correction at 550 nm.

### 2.11 Statistical analysis

All data are expressed as mean ± standard error of the mean (SEM). A Kolmogorov–Smirnov test was performed to determine parametric distribution of the data. Statistical differences of parametric data were analyzed using one-way ANOVA, while nonparametric data were analyzed with a Kruskal–Wallis test for comparisons among three or more groups, followed by a Tukey's post hoc test. The data were analyzed using GraphPad Prism (v. 8.00; GraphPad Software Inc, La Jolla, USA). A p-value <0.05 was considered statistically significant (*p < 0.05, **p < 0.01, and ***p < 0.001).

## 3 Results

### 3.1 Activated human PSCs preserve human CM β-cells mitochondrial activity in co-culture

PSCs are tissue endogenous MSCs of the pancreas [20]. Quiescent PSCs (qPSC) express several intermediate filament proteins such as desmin, glial fibrillary acidic protein (GFAP), and vimentin. Culture-adapted PSCs undergo activation during which they experience morphological and cytological changes and gain a myofibroblastic phenotype due to α-SMA expression and ECM production [21]. The human PSCs (HPSCs) we used were first characterized for the following PSCs markers: α-SMA, vimentin, desmin and GFAP. Strong cytoplasmic positivity for α-SMA and vimentin was observed by immunofluorescence, while desmin and GFAP showed mild positive staining (Fig. S1). The high expression level of α-SMA in HPSCs suggests that the primary HPSCs were activated [14,22].

**Fig. 1:**
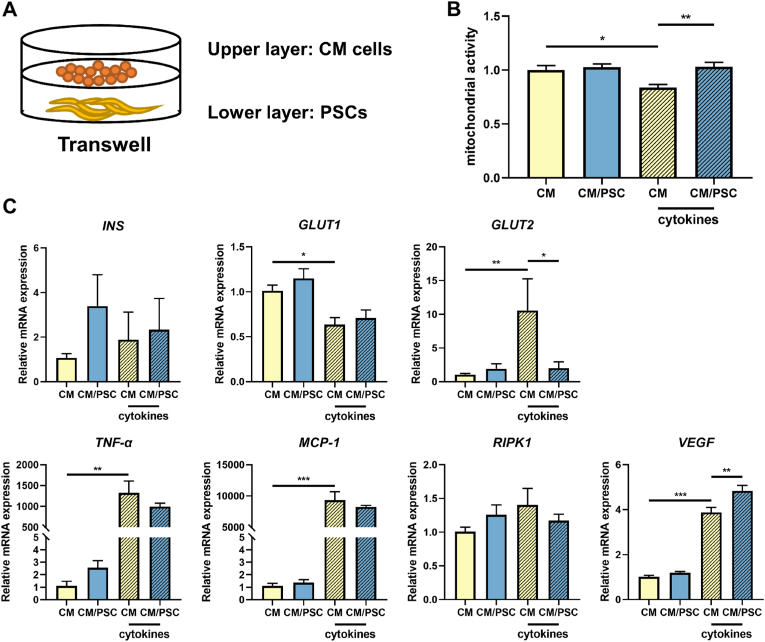
**Mitochondrial activity and gene expression levels in CM β-cells in presence and absence of the cytokine mix of IFN-γ, TNF-α and IL-1β**. **(A)** Schematic illustration of the co-culture setting of CM cells with HPSCs in Transwell systems. **(B)** Mitochondrial activity in CM β-cells. **(C)** Gene expression levels of *INS*, *GLUT1*, *GLUT2*, *TNF-α*, *MCP-1*, *RIPK1* and *VEGF* by qPCR in CM β**-**cells. Results represent mean ± SEM (n = 6). The statistical differences were quantified using one-way ANOVA analysis with Tukey's post hoc test (*p < 0.05, **p < 0.01, ***p < 0.001).

Subsequently, we investigated the impact of human PSC on β-cells. Human CM β-cells were cultured in RPMI1640 medium, in which we gradually decreased glucose concentrations until reaching 0.8–1.0 mM glucose to achieve a higher capacity to respond to glucose stimulation [17]. This procedure of gradually lowering glucose levels enables CM β-cells to respond stronger with insulin secretory genes upon glucose stimulation (as indicated in 2.2). The β-cells were subsequently cultured with activated HPSCs in Transwells for 24 h (Fig. 1A). Cytokine-induced stress was induced by incubating the cells with a cytokine cocktail of human IFN-γ (2000 U/mL), TNF-α (2000 U/mL), and IL-1β (150 U/mL). To study the protective effects of co-culture with HPSCs on cytokine-induced stress in CM β-cells, we tested the mitochondrial activity of CM cells by applying a WST-1 assay. As shown in Fig. 1B, in the absence of cytokine stress, co-culture with HPSCs did not change the mitochondrial activity of human β-cells. Exposure to cytokines reduced the mitochondrial activity of CM cells by 16.2 ± 8.8 % (p < 0.05), which was prevented by co-culturing the β-cells with HPSCs (p < 0.01).

Next, we tested the impact of cytokines on CM β-cells and the potential rescuing effects of HPSCs on these cells. We assessed genes related to insulin secretion, as the CM β-cells do not secrete insulin upon glucose stimulation. To this end, we tested the expression of β-cell genes related to insulin secretory capacity *i.e. INS*, *GLUT1*, *GLUT2* when the cells were co-cultured with HPSCs in presence and absence of cytokine stress. Additionally, we tested the potential rescuing effects of HPSCs on inflammation-related genes (*TNF-α*, *MCP-1*), the necroptosis related gene *RIPK1* and the angiogenesis-related gene *VEGF* (Fig. 1C). As shown in Fig. 1C, the expressions of *INS* and *RIPK1* were not affected by the exposure to the cytokines nor by co-culture with HPSCs. However, cytokine exposure downregulated the expression of *GLUT1* to 63.8 ± 18.5 % (p < 0.05) of non-cytokine exposed control β-cells. Cytokine exposure enhanced *GLUT2*, *TNF-α*, *MCP-1* and *VEGF* expression by 10.6-fold (p < 0.01), 1328-fold (p < 0.01), 9329-fold (p < 0.001) and 3.9-fold (p < 0.001) respectively. Co-culture with HPSCs attenuated the cytokine-induced increase in *GLUT2* (1.9-fold, p < 0.05), while an even higher *VEGF* level (4.8-fold, p < 0.01) was observed.

The effect of co-culturing with HPSCs in the absence or presence of cytokine stress was also investigated in CM cells cultured at high glucose levels (11 mM, Fig. S2). In general gene expression in CM β-cells exposed to high-glucose concentrations (11 mM) were decreased compared to CM β-cells cultured at lower glucose concentrations. The mitochondrial activity of CM cells cultured in high glucose was not affected by coculturing with HPSCs in absence of cytokine stress. This was different when the cells were exposed to the cytokine mix. In presence of cytokines, the mitochondrial activity decreased in the absence of HPSCs in CM β-cells to 47.3 ± 1 % and with co-culture of HPSCs to 59.1 ± 2 % compared to control group (both p < 0.001). Co-culturing with HPSCs prevented the cytokine-induced decrease in mitochondrial activity by 11.8 % (p < 0.001). CM β-cells cultured in 11 mM glucose did not express *INS* or *GLUT2*. Cytokine exposure upregulated the gene expression levels of *GLUT1*, *TNF-α*, *MCP-1* and *VEGF* by 1.5-fold, 1853-fold, 7514-fold and 5.0-fold respectively (p < 0.01, p < 0.001, p < 0.001, and p < 0.001) in CM β-cells in the absence of HPSCs. Co-culture with HPSCs under cytokine exposure enhanced the expression of *GLUT1*, *MCP-1* and *VEGF* by 1.7-fold, 9105-fold, and 5.7-fold (p < 0.05, p < 0.001, p < 0.05) and downregulated the expression of *TNF-α* to 0.8-fold (p < 0.05) compared to CM β-cells only.

### 3.2 Activated HPSCs preserved human islet mitochondrial activity

Subsequently, we investigated the impact of HPSC co-culture on human islets in the presence and absence of cytokine-induced stress. Isolated human islets were cultured alone (Islets) or co-cultured with HPSCs either in Transwell systems (Islets/HPSC Transwell) or with direct contact (Islets/HPSC contact). This approach allowed us to determine whether HPSCs support islet cells in a paracrine fashion in the absence of direct contact with HPSCs or with direct contact. The test was done with and without stimulation with the cytokine cocktail. The group with human islets alone was used as control.

**Fig. 2:**
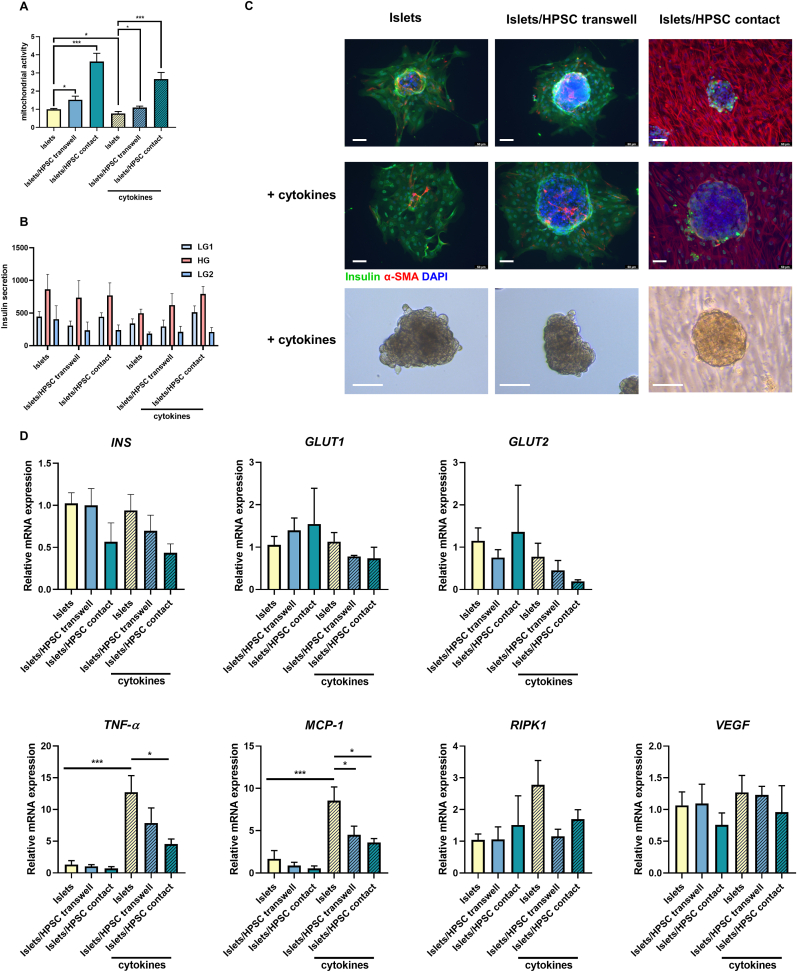
**The co-culture of HPSCs preserved human islet mitochondrial activity *in vitro* in presence and absence of IFN-γ, TNF-α and IL-1β**. **(A)** Mitochondrial activity of human islets as quantified by the WST-1 assay after 24h culture under normal conditions or after cytokine exposure. **(B)** GSIS and **(C)** insulin staining was applied to indicate insulin secretion levels. A light microscope image is shown to indicate the islet morphology which remain intact during the experiment. Scale bar is 50 μm. **(D)** Gene expression levels of *INS*, *GLUT1*, *GLUT2*, *TNF-α*, *MCP-1*, *RIPK1* and *VEGF* in human islets by qPCR. LG, low glucose, 2.75 mM; HG, high glucose, 16.7 mM. Insulin secretion is expressed as mU L^−1^ μg^−1^ DNA hour^−1^. Results represent mean ± SEM (n = 4). The statistical differences were quantified using one-way ANOVA analysis with Tukey's post hoc test (*p < 0.05, **p < 0.01, ***p < 0.001).

In absence of cytokine stress, we found that the co-culture with HPSCs significantly upregulated mitochondrial function by 1.5-fold (p < 0.05) in the Transwell system and 3.6-fold (p < 0.001) with direct contact (Fig. 2A). Cytokine exposure reduced the mitochondrial activity in islets by 23.3 ± 11.2 % (p < 0.05). This reduction was attenuated by co-culturing the islets with HPSCs, preventing the cytokine-induced loss by 32.8 % (p < 0.05) in Transwells and 189 % (p < 0.001) with direct contact with HPSC. The morphology of human islets didn't show much modification under cytokine exposure (Fig. 2C).

The effect of HPSC co-culture on human islets was also studied by quantifying glucose induced insulin secretion (GSIS), immunofluorescent staining for insulin and related gene expression (*INS*, *GLUT1*, *GLUT2*) (Fig. 2B–D). However, no significant differences were found in insulin secretion or the aforementioned related genes when human islets were cultured with HPSCs (Fig. 2D).

The gene expression levels of *TNF-α*, *MCP-1, RIPK1* and *VEGF* in human islets were also determined (Fig. 2D). After exposure to cytokines, the expression was increased by 12.7-fold for *TNF-α* (p < 0.001) and 8.6-fold for *MCP-1* (p < 0.001). The islets/HPSC Transwell group had a 47 % (p < 0.05) lower expression of *MCP-1* in human islets. Islets/HPSC contact group decreased the expression of *TNF-α* by 64 % (p < 0.05) and the cytokine-induced expression of *MCP-*1 by 58 % (p < 0.05). We did not find any change in cytokine-induced gene expression of *RIPK1* or *VEGF*. Also, co-culture with HPSC did not change this expression.

### 3.3 Activated HPSCs display altered gene expression levels after cytokine stimulation and is dependent on both direct cellular interaction and on paracrine effects

**Fig. 3:**
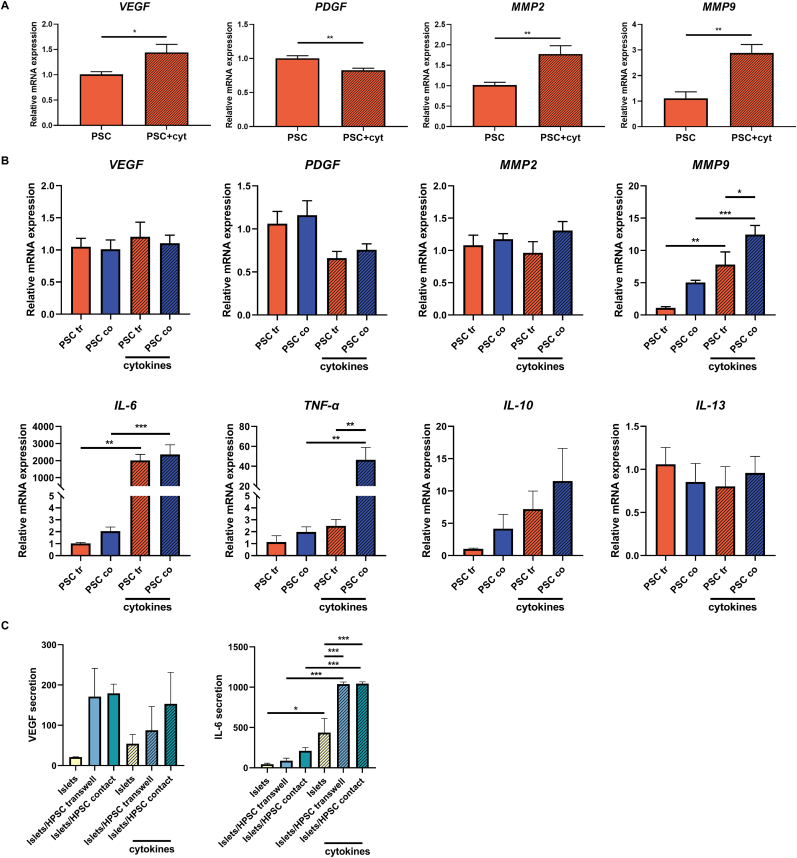
**Activated HPSCs displayed altered gene expression levels by IFN-γ, TNF-α and IL-1β stimulation and was dependent on the applied co-culture system. (A)** Gene expression levels of *VEGF*, *PDGF*, *MMP2* and *MMP9* in HPSCs co-cultured with CM β-cells in Transwell systems in presence and absence of cytokine exposure by qPCR. **(B)** Gene expression levels of *VEGF*, *PDGF*, *MMP2*, *MMP9*, *IL-6*, *TNF-α*, *IL-10* and *IL-13* in HPSCs co-cultured with human islets either in Transwell systems or with direct contact either in the presence and absence of cytokine exposure by qPCR. **(C)** VEGF and IL-6 secretion (pg/mL) in human islet-only group or co-culture with PSCs by ELISA. Results represent mean ± SEM (n = 4). The statistical differences were quantified using one-way ANOVA analysis with Tukey's post hoc test (*p < 0.05, **p < 0.01, ***p < 0.001).

To determine potential bioactive factors derived from HPSCs that might be responsible for the protective role of HPSCs on cytokine-induced dysregulation of human CM β-cells and human islets, we tested the expression of several candidate genes that might be involved in beneficial or inflammatory effects [23,24]. These included VEGF, PDGF, MMP2, MMP9, IL-6, TNF-α, IL-1Ra, IL-10, IL-4 and IL-13 using qPCR (Fig. 3). VEGF and PDGF are key actors in the regeneration of damaged pancreatic tissue [25,26], while MMP2 and MMP9 are essential for ECM remodeling [27,28]. IL-6, TNF-α, IL-4 and IL-13 are proinflammatory cytokines and IL-1Ra and IL-10 are regulatory cytokines.

First, we studied PSCs in a Transwell setting exposed to CM β-cells before conducting studies on human islets using both Transwell systems and assays allowing direct contact between HPSCs and human islets. There were differences in gene expression in PSCs in presence and absence of cytokines when the stromal cells were co-cultured with CM β-cells in the Transwell systems. Cytokine exposure to PSCs in a Transwell setting at low glucose (Fig. 3A) increased the expression of *VEGF* by 1.4-fold (p < 0.05) and decreased the expression of *PDGF* by 0.8-fold (p < 0.01) compared to PSCs co-cultured with CM β-cells in the absence of cytokines. The expression levels of *VEGF* and *PDGF* were also tested in Transwell-cultured PSCs with CM β-cells in medium with high glucose (Fig. S2C). Here, at high-glucose levels, the expression of *PDGF* was decreased by cytokine exposure. Cytokine exposure to PSCs co-cultured CM β-cells setting at low glucose significantly elevated the expression levels of *MMP2* and *MMP9* to 1.8-fold (p < 0.01) and 2.9-fold (p < 0.01) respectively, compared to those without cytokines (Fig. 3A). However, in CM β-cells cultured in high glucose conditions, cytokine exposure elevated the expression of *MMP9* by 6.4-fold (p < 0.001) but did not affect the expression of *MMP2* (Fig. S2C). For technical reasons, we could not perform the same analysis with PSCs that were in direct contact with CM β-cells.

Next, human islets were co-cultured with PSCs either in Transwell systems or with direct cellular contact. In Transwell-cultured PSCs with human islets, no significant difference in gene expression of *VEGF* and *PDGF* was detected after cytokine stimulation (Fig. 3B). Also, with direct contact between PSCs and human islets we found no significant changes in *VEGF* and *PDGF*. In the Transwell setting, treatment with cytokines did not change the expression of *MMP2* in PSCs. For *MMP9*, cytokine exposure significantly increased its expression in the Transwell setting (7.8-fold, p < 0.01). In the direct contact co-culture group, we found a 1.6-fold higher *MMP9* expression in PSCs than that in Transwell group (p < 0.05) under cytokine exposure.

The expression of inflammation-related genes (*IL-6*, *TNF-α*, *IL-1Ra*, *IL-10*, *IL-4* and *IL-13*) was also investigated in PSCs using qPCR (Fig. 3B). *IL-1Ra* and *IL-4* was not expressed in HPSCs, independent of the culture system applied. In a Transwell setting, cytokine exposure significantly increased the *IL-6* expression in PSCs by 2011-fold (p < 0.01) in PSCs. When direct cellular contact was allowed, the increase was lower but still significant at 1152-fold (p < 0.001). Cytokine exposure also enhanced the *TNF-α* expression in HPSCs by 23.5-fold (p < 0.01) in the Transwell system. *TNF-α* expression was also significantly higher in the PSCs co-culture group, elevated by 18.7-fold under cytokine stimulation (p < 0.01).

To further study the secretion of VEGF and IL-6 on a protein level in different culture conditions, we applied an ELISA test to determine the secretion of these two factors in supernatant of different groups (Fig. 3C). We did not find statistically significant differences in VEGF secretion between the cytokine-exposed groups or in islets co-cultured with HPSCs. However, we observed a significantly elevated cytokine-induced IL-6 secretion in the islet-only group, Islets/HPSC Transwell and Islets/HPSC contact groups. The elevation in IL-6 secretion was 9.7-fold (p < 0.05), 11.7-fold (p < 0.001) and 5.0-fold (p < 0.001), respectively. The secretion levels of IL-6 in the Islets/HPSC Transwell and Islets/HPSC contact group were higher than in islets-only group by 2.1-fold (p < 0.001) and 4.9-fold (p < 0.001) under cytokine exposure.

### 3.4 Microencapsulation of human islets in composite capsules together with PSC co-culture enhance islet viability and survival

**Fig. 4:**
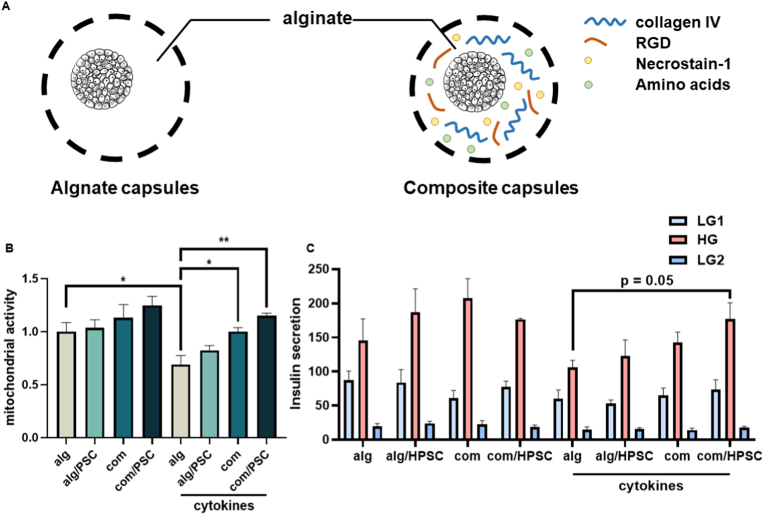
**Microencapsulation of human islets in composite capsules together with PSC co-culture enhanced islet viability and survival.** (A) Schematic representation of alginate-based microcapsules with a regular alginate-calcium design and a composite design containing collagen type IV/RGD, Necrostatin-1 and amino acids (alanine, glutamine, glycine). (B) Mitochondrial activity in presence and absence of IFN-γ, TNF-α and IL-1β and (C) insulin secretion function in the presence and absence of IFN-γ, TNF-α and IL-1β of human islets when microencapsulated in capsules with alginate or composite designs, either co-cultured with PSCs or not. Cytokine cocktails caused the stimulation. Results represent mean ± SEM (n = 4). The statistical differences were quantified using one-way ANOVA analysis with Tukey's post hoc test (*p < 0.05, **p < 0.01, ***p < 0.001).

Next, we investigates whether the PSCs provide benefits for islets in alginate microcapsules, as these islets suffer from cytokine stress post-transplantation, which is associated with up to 60 % loss of the islet tissue in the first two weeks after implantation [7,29]. We tested the impact of HPSCs in conventional alginate capsules [12,30], as well as in recently developed composite capsules containing the immunomodulatory molecule pectin and ECM components (collagen type IV and RGD) with necrostatin-1 (Nec-1), as well as amino acids (alanine, glutamine, glycine) [9,31]. To test the impact of HPSCs in these capsules, we compared the following groups (Fig. 4A): human islets encapsulated in alginate (alg: control group) or a composite group (com) of collagen type IV and RGD with Nec-1and amino acids [9]. Encapsulated human islets were co-cultured with PSCs (alg/PSC and com/PSC) or alone. HPSCs were cultured as monolayer followed by the addition of encapsulated human islets for co-culture. They were cultured in the absence and presence of the human cytokine cocktail. After 24 h, the mitochondrial activity of human islets in control capsules or composite capsules were tested by WST-1, and we tested the glucose-induced insulin secretion by GSIS.

Cytokine exposure decreased mitochondrial activity in the alg group with 31 % (p < 0.05), which was reversed in the com group (p < 0.05) and com/PSC group (p < 0.01, Fig. 4B). The HPSCs seem to have a synergistic effect on islet viability, but this only reached statistically significant levels when compared to the human islets in alginate capsules (com vs. alg, p < 0.05 and com/PSC vs. alg, p < 0.01). No significant differences were found in GSIS results, though p = 0.05 between the alg group and com/PSC group when comparing insulin secretion under high glucose condition.

## 4 Discussion

The loss of isolated pancreatic islets in the immediately after implantation is caused by inflammatory stress associated with implantation surgery and is exacerbated by increased susceptibility in β-cells to cytokine stress due to damage to the ECM during the isolation of islets from the pancreas [7]. This loss of islet viability during islet isolation in the post-transplantation period represents a significant obstacle to the success of islet transplantation [32]. Accumulating evidence indicates that this loss of islet cells might be prevented by co-culturing or co-transplantation of tissue-resident stromal cells and culture-adapted MSCs [33,34]. Sharing the same mesodermal progenitors, PSCs have been considered for their possible role in preserving islet engraftment, function and outcomes after transplantation. Recent studies have initiated an exploration of the potential involvement of PSCs in the interactions with β-cells [35,36]. However, limited progress has been made in yielding substantial results. Previous studies have shown that PSCs may facilitate the function of β-cell [35], similar to the way they fuel the survival, proliferation and prevent the apoptosis of pancreatic tumor cells. Here, we showed that human PSCs can rescue β-cell viability under cytokine-induced stress. Furthermore, PSCs reduced inflammatory stress in both β-cell and human islets, proving the direct protective effects of PSCs on β-cells. This protective effect against cytokine-induced stress might depend on the alteration of expression of ECM-related and inflammation-related genes.

Culture-adapted PSCs undergo canonical endothelial-mesenchymal transition (EMT), through expressing EMT markers such as α-SMA and other ECM proteins [35]. The potential protective role of activated PSCs in supporting β-cell viability and function involves a complex interplay within the pancreatic microenvironment via three potential mechanisms. First, PSCs are known to secrete various trophic factors and cytokines that create a supportive milieu for β-cell survival and function, such as VEGF and PDGF [23,24]. PDGF promotes β-cell proliferation by activating downstream signaling and inducing cell cycle gene expression through PDGF receptor expressed on β-cells [26]. This is why we applied Transwell systems to allow us to study possible paracrine effects of PSCs on human β-cells. Second, PSCs contribute to downregulation of inflammation, mitigating the detrimental effects of these processes in β-cells. Islets suffer from various stresses during the isolation process such as from enzymatic digestion and loss of vascular support. Consequently, upon culture or implantation, they become vulnerable to oxidative, hypoxic, nutrient shortage and proinflammatory cytokines [37,38]. Damaged β-cells under cytokine stress can generate danger signals such as inflammatory cytokines that induce nearby cell necroptosis and apoptosis [33,38]. PSCs might mitigate all these processes by suppressing inflammation. Third, emerging evidence suggests that PSCs play a role in modulating the ECM composition, influencing the structural integrity of the islets and promoting a conducive environment for β-cell health. Two metalloproteinases, MMP2 and MMP9, inhibit β-cell apoptosis [39], which was enhanced by cytokine exposure in PSCs [40]. Overall, the protective role of PSCs in preserving β-cell viability appears to be multifaceted, involving both paracrine signaling and physical interactions within the pancreatic microenvironment. Notably however our data do show that impact of PSCs is determined by the glycemic state in which most beneficial effects were found under normal glucose conditions and to a lesser extend under hyperglycemia, as the comparative mitochondrial activity of CM cells in low-glucose conditions under cytokine exposure in either CM only group or co-culture with PSCs was higher than those in high-glucose conditions.

The interaction between PSCs and human islets has been a subject of interest in research, particularly in the context of understanding the microenvironment of the pancreas and its impact on islet function. The Transwell co-culture system was applied to investigate how PSCs influence human islets through paracrine effects. In Transwell co-culture setups, we noticed that human islets maintained their mitochondrial activity when co-cultured with activated PSCs, especially under cytokine exposure. This observation indicates a possible paracrine signaling pathway through which PSCs aid human β-cells. This seems to be accomplished by the unaffected VEGF, PDGF and TNF-α levels in PSCs under cytokine exposure, as well as the attenuated chemokine MCP-1 expression. However, direct contact between PSCs and human islet cells also demonstrated improved mitochondrial activity. This was somewhat surprising, as in the direct interaction studies, we found upregulation of the proinflammatory cytokines TNF-α and a similar enhanced IL-6 expression.

Research on PSCs has predominantly been focused on their role in pathogenesis of pancreatic cancer and chronic pancreatitis [15,41]. Under culture adaption, PSCs become activated and possess a proliferative, migratory phenotype, inducing desmoplasia by synthesizing abundant ECM components, such as collagens, fibronectin, laminin, and hyaluronic acid, and unbalanced expression of MMPs and tissue inhibitors of metalloproteinases (TIMPs). Laminin and its receptors may underlie the cytoprotective effects of PSCs [13,42]. The laminin secreted by PSCs, together with laminin sequence RGD, further enhanced the interaction between PSCs/islets to support islet health. This likely explains for the enhanced beneficial effects we found in the composite capsules that contained RGD in addition to collagen type IV.

The effects of PSCs on mitochondrial activity in β-cells and human islets under cytokine-induced stress are significant and multifaceted. Mitochondria play a critical role in maintaining cellular energy homeostasis, and their function is crucial for β-cell viability and insulin secretion. Proinflammatory cytokines, such as IFN-γ, TNF-α and IL-1β, can have several inhibitory effects on mitochondrial function, including electron transport chain inhibition, mitochondrial membrane potential loss, ATP production reduction among other processes [43,44]. Our study demonstrated that co-culturing β-cells with PSCs, especially under inflammatory conditions, helps to preserve mitochondrial activity. This preservation is likely due to several factors, including the secretion of trophic factors and cytokines by PSCs, which create a supportive microenvironment for β-cells. VEGF and PDGF, for instance, are known to enhance mitochondrial function and promote cell survival [45,46]. Moreover, the attenuation of inflammatory responses by PSCs reduces the detrimental impact of cytokines on mitochondrial integrity. In the presence of PSCs, the expression of chemokines like MCP-1 is reduced, which helps to mitigate mitochondrial stress and prevent apoptosis [47,48]. Interestingly, our observations also noted improved mitochondrial activity in direct contact setups. This suggests that PSCs might exert a complex protective effect that balances proinflammatory signals by production of essential survival factors. Overall, the interaction between PSCs and β-cells appear to stabilize mitochondrial function, enhancing cellular resilience against cytokine-induced damage.

Our data suggest that PSCs, typically depleted during pancreatic islet isolation, may be valuable in preserving islet viability and should be considered as accessory cells for co-transplantation with islets, with or without an immunoisolating capsule. These PSCs can be harvested concomitantly with pancreatic islets. A parallel approach, akin to strategies employed with MSCs, involves exploring PSCs' potential in secretion of cytokines or exosomes, an aspect that has garnered substantial attention recently [23,49,50]. In our current study we also demonstrated the beneficial effects of PSCs on microencapsulated human islets via paracrine effects. The effects were more pronounced on the composite microcapsules than on islets in the conventional alginate-capsules. Modification of microcapsules enables encapsulated islets to sustain the danger-associated molecular pattern (DAMP)-induced immune activation [8,51]. The pectin with low degree of methyl esterification (DM) had profound influence on the performance of islets in capsules [8,10]. The composite design capsules, with collagen type IV, laminin sequence RGD, Nec-1, and amino acids (alanine, glutamine, glycine) together with DM18 pectin, provide a superior microenvironment for islet transplantation with immunoisolation [9]. The combination of composite capsules together with co-transplantation with PSCs likely synergistically prolong the life of islet-graft.

This study embarks on an exploration of the supportive role played by PSCs in sustaining the viability of human pancreatic β-cells *in vitro*. Understanding the molecular basis of this protective effect may offer insights into the factors influencing islet resilience under stressful conditions. Such insights could be crucial for developing strategies to improve islet survival during transplantation or in conditions associated with inflammation. Additionally, we extend our inquiry to the realm of immunoisolated islets, investigating how PSCs contribute to the resilience of transplanted islets, together with composite microcapsule design, when confronted with the challenges posed by inflammatory cytokines. Exploring the mechanisms behind this synergy may uncover insights into how physical and biochemical cues from the microenvironment interact to promote islet survival. This knowledge has the potential to inform the development of advanced biomaterials and co-culture approaches, thereby advancing the field of islet transplantation.

In conclusion, we demonstrate a multifaceted role for activated PSCs in supporting pancreatic β-cell health and islet survival. Further investigations into the underlying molecular mechanisms and their implications could pave the way for innovative interventions to enhance pancreatic islet function and improve the success of islet transplantation therapies.

## CRediT authorship contribution statement

**Tian Qin:** Writing – review & editing, Writing – original draft, Investigation, Data curation, Conceptualization. **Shuxian Hu:** Writing – review & editing, Methodology. **Defu Kong:** Writing – review & editing, Methodology. **Jonathan R.T. Lakey:** Writing – review & editing, Supervision. **Paul de Vos:** Writing – review & editing, Writing – original draft, Project administration, Funding acquisition.

## Declaration of competing interest

The authors declare that they have no known competing financial interests or personal relationships that could have appeared to influence the work reported in this paper.

## Data Availability

Data will be made available on request.
