# Supplementary material for: Pancreatic stellate cells support human pancreatic β-cell viability in vitro and enhance survival of immunoisolated human islets exposed to cytokines
Source: Mater Today Bio. 2024 Jun 18;27:101129. doi: 10.1016/j.mtbio.2024.101129 (PMC11253154; doi:10.1016/j.mtbio.2024.101129)

**Pancreatic stellate cells support human pancreatic β-cell viability in vitro and enhance survival of human immunoisolated islets exposed to cytokines**

Tian Qin ^a*^, Shuxian Hu ^a, b^, Defu Kong ^c^, Jonathan R.T. Lakey ^d, e^, Paul de Vos ^a^

^a^ Immunoendocrinology, Division of Medical Biology, Department of Pathology and Medical Biology, University of Groningen and University Medical Center Groningen, Hanzeplein 1, EA 11, 9713 GZ, Groningen, The Netherlands

^b^ Biological and Environmental Engineering, Cornell University, Ithaca, NY 14853, USA

^c^ Department of Liver Surgery, Renji Hospital, School of Medicine, Shanghai Jiao Tong University, Shanghai 200127, China.

^d^ Department of Surgery, University of California Irvine, Irvine, CA 92868, USA

^e^ Department of Biomedical Engineering, University of California, Irvine, Irvine, CA 92697, USA

**Supplementary tables and figures**

**Table S1.** The list of antibodies used in this study.

| **Protein** | | **Company** | | **Lot no.** |
| --- | --- | --- | --- | --- |
| Insulin | | | Cell Signaling Technology | #3014 |
| α-SMA | | | Sigma Aldrich | A5228 |
| Desmin | | | Abcam | ab8592 |
| GFAP | | | Cell Signaling Technology | #3670 |
| Vimentin | | | Cell Signaling Technology | #5741 |
| CK19 | | | Abcam | ab52625 |
| CD68 | | | Abcam | ab125212 |
| Goat anti-Rabbit Secondary Antibody, Alexa Fluor^TM^ 488 | | | Invitrogen | A-11008 |
| Goat anti-Mouse Secondary Antibody, Alexa Fluor^TM^ 555 | | | Invitrogen | A-21422 |
|  |  |  | | |

**Table S2.** Primer sequences for qRT-PCR.

| **Gene** | **Species** | **Forward Sequence 5′-3′** | **Reverse Sequence 5′-3′** |
| --- | --- | --- | --- |
| *GAPDH* | Human | CAAATTCCATGGCACCGTCAA | AGCATCGCCCCACTTGATTT |
| *INS* | Human | CTAGTGTGCGGGGAACG | CACGCTTCTGCAGGGAC |
| *GLUT1* | Human | TCATCGTGGCTGAACTCTTC | GATGAAGACGTAGGGACCAC |
| *GLUT2* | Human | TGCTGTCTCTGTATTCCTTGTG | TGCTCACATAACTCATCCAAGAG |
| *TNF-α* | Human | GAGGCCAAGCCCTGGTATG | CGGGCCGATTGATCTCAGC |
| *MCP-1* | Human | GAAAGTCTCTGCCGCCCTT | GGTGACTGGGGCATTGATTG |
| *RIPK1* | Human | AGCTCCTGGGCGTCATCATA | AGGTCTGCGATCTCGGCTTT |
| *VEGF* | Human | AGGAGGAGGGCAGAATCATCA | CTCGATTGGATGGCAGTAGCT |
| *PDGF* | Human | CAGCGACTCCTGGAGATAGAC | ATGCTTAGTGGCATGGACCC |
| *MMP2* | Human | TGTGTTGTCCAGAGGCAATG | ATCACTAGGCCAGCTGGTTG |
| *MMP9* | Human | CGAACTTTGACAGCGACAAG | CACTGAGGAATGATCTAAGCCC |
| *IL-6* | Human | ACTCACCTCTTCAGAACGAATTG | CCATCTTTGGAAGGTTCAGGTTG |
| *IL-10* | Human | TCAAGGCGCATGTGAACTCC | GATGTCAAACTCACTCATGGCT |
| *IL-13* | Human | CCTCATGGCGCTTTTGTTGAC | TCTGGTTCTGGGTGATGTTGA |

**Figure S1.** Expression of characteristic PSC markers (α-SMA, vimentin, desmin and GFAP) in primary human PSCs. Scale bar is 50 μm.


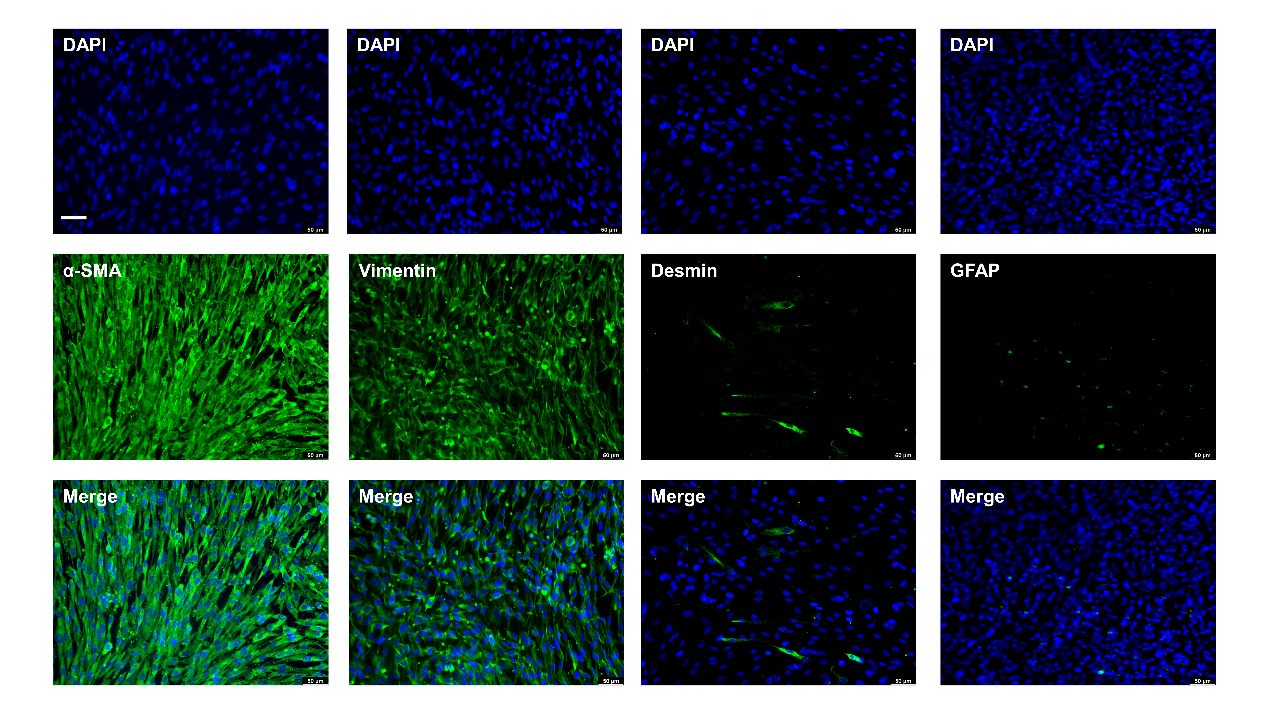


**Figure S2.** Mitochondrial activity **(A)** and gene expression levels in **(B)** CM cells cultured in 11 mM and **(C)** HPSCs.


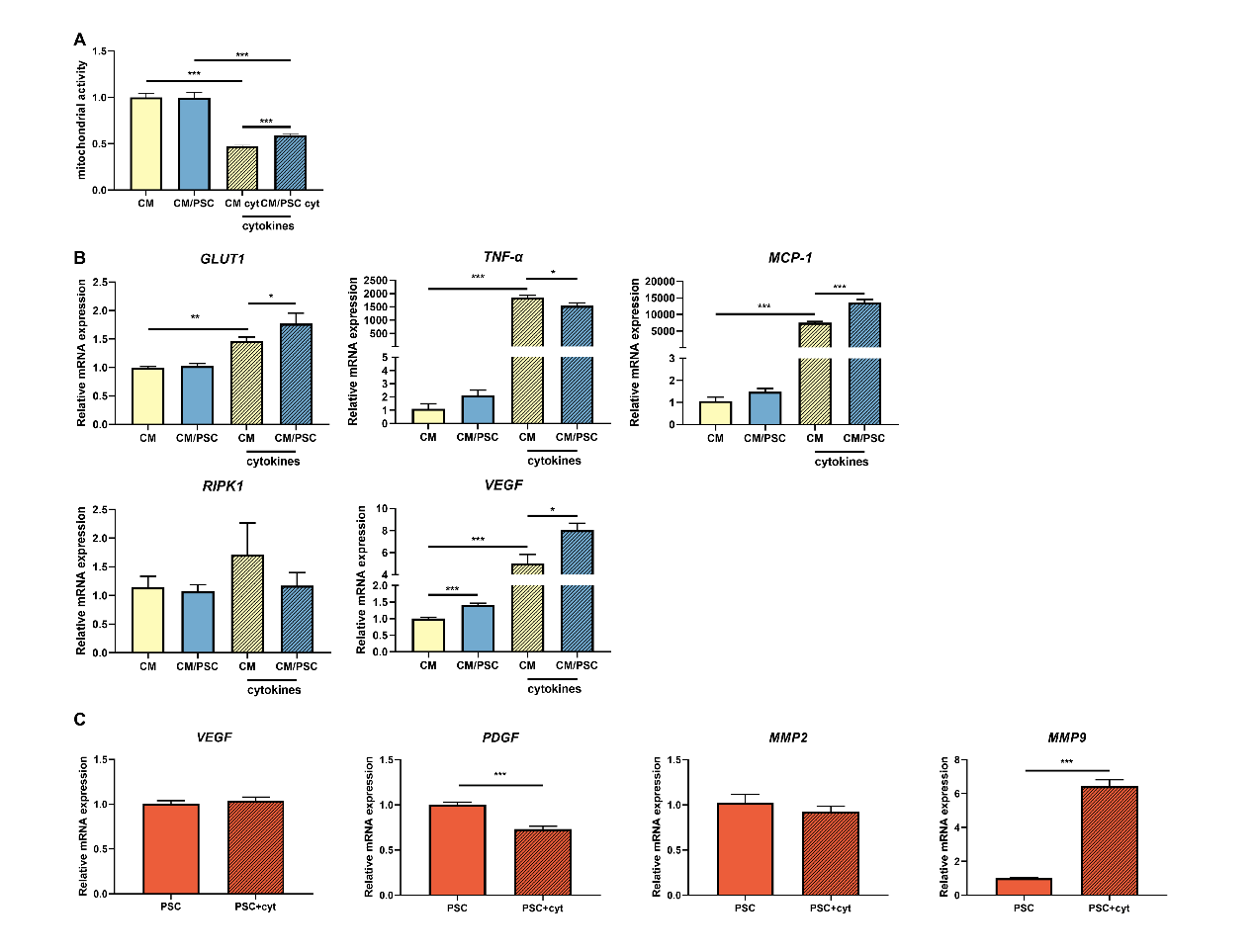

Supplement: Multimedia component 1 [file mmc1.docx]
